# Transforming Industrial Manipulators via Kinesthetic Guidance for Automated Inspection of Complex Geometries

**DOI:** 10.3390/s23073757

**Published:** 2023-04-05

**Authors:** Charalampos Loukas, Momchil Vasilev, Rastislav Zimmerman, Randika K. W. Vithanage, Ehsan Mohseni, Charles N. MacLeod, David Lines, Stephen Gareth Pierce, Stewart Williams, Jialuo Ding, Kenneth Burnham, Jim Sibson, Tom O’Hare, Michael R. Grosser

**Affiliations:** 1 SEARCH: Sensor Enabled Automation, Robotics & Control Hub, Centre for Ultrasonic Engineering (CUE), Department of Electronic & Electrical Engineering, University of Strathclyde, Royal College Building, 204 George Street, Glasgow G1 1XW, UK; momchil.vasilev@strath.ac.uk (M.V.); rastislav.zimermann@strath.ac.uk (R.Z.); randika.vithanage@strath.ac.uk (R.K.W.V.); ehsan.mohseni@strath.ac.uk (E.M.); charles.macleod@strath.ac.uk (C.N.M.); david.lines@strath.ac.uk (D.L.); s.g.pierce@strath.ac.uk (S.G.P.); 2 Welding Engineering and Laser Processing Centre, University of Cranfield, Cranfield MK43 0AL, UK; s.williams@cranfield.ac.uk (S.W.);; 3 Digital Factory, NMIS, Industrial Business Park, Renfrew PA4 8BE, UK; kenneth.burnham@strath.ac.uk; 4 Babcock International Group PLC, Bristol BS16 1EJ, UK; jim.sibson@babcockinternational.com; 5 Spirit Aerosystems Belfast, Belfast BT3 9DZ, Northern Ireland, UK; tom.ohare@spiritaero.com; 6 Spirit Aerosystems, Wichita, KS 67210, USA

**Keywords:** collaborative robotics, non-destructive evaluation, WAAM, kinesthetic, robot programming

## Abstract

The increased demand for cost-efficient manufacturing and metrology inspection solutions for complex-shaped components in High-Value Manufacturing (HVM) sectors requires increased production throughput and precision. This drives the integration of automated robotic solutions. However, the current manipulators utilizing traditional programming approaches demand specialized robotic programming knowledge and make it challenging to generate complex paths and adapt easily to unique specifications per component, resulting in an inflexible and cumbersome teaching process. Therefore, this body of work proposes a novel software system to realize kinesthetic guidance for path planning in real-time intervals at 250 Hz, utilizing an external off-the-shelf force–torque (FT) sensor. The proposed work is demonstrated on a 500 mm^2^ near-net-shaped Wire–Arc Additive Manufacturing (WAAM) complex component with embedded defects by teaching the inspection path for defect detection with a standard industrial robotic manipulator in a collaborative fashion and adaptively generating the kinematics resulting in the uniform coupling of ultrasound inspection. The utilized method proves superior in performance and speed, accelerating the programming time using online and offline approaches by an estimate of 88% to 98%. The proposed work is a unique development, retrofitting current industrial manipulators into collaborative entities, securing human job resources, and achieving flexible production.

## 1. Introduction

The growth of High-Value Manufacturing (HVM) sectors, such as defense, nuclear, aerospace, and marine, comes with a need to fabricate and inspect components with ever-increasing structural complexity and volume. This emerging industrial demand coupled with the high-reliability and precision demands in those sectors has grown their desire to adopt and integrate automated robotic solutions in their production cycle. Alongside the prevalence of Industry 4.0, paired with the latest technological paradigms, smart factories capable of delivering products with highly customized geometry and quality specifications are being sought [1]. To meet the demand surge, flexible manufacturing approaches must be considered with characteristics such as:

1. Intuitive robotic path planning for complex geometries;
2. Adaption and interaction of the process per component variations;
3. Deployment and utilization of big data analysis tools for process optimization.

An important aspect to realize flexible automation is in the approach of robotic programming that generates motion path planning. As robotic path teaching is a vital task for all robotic applications, the efficiency of the programming method directly affects the production cycle. Online programming (OP) is predominantly used to manually teach, where the input of a trained operator is required to teach the path points in space through a teach pendant [2,3]. This lead-through method requires robotics knowledge to generate kinematics and awareness of possible collision singularities during trajectory planning. Although OP is characterized as a time-consuming method with an unfavorable ratio between programming and production time, it is still used predominantly by Small-to-Medium Enterprises (SMEs) [2,3]. This prevalent method has drawbacks, such as mandatory downtime, as the robot cannot be utilized during programming, and the generated program lacks flexibility, requiring re-programming to accommodate for even slight changes in the same workpiece. On the other hand, Off-Line Programming (OLP) relies on the availability of accurate Computer-Aided Design (CAD) of the workpiece and the operational robotic cell. Although OLP proves superior for large-volume production, it still requires a high level of operator expertise and generates tedious programming overhead. However, the workload is shifted to a software engineer, minimizing downtime as programming can be achieved alongside production, incorporating robotic simulation. This functionality can increase production efficiency, especially through Computer-Aided Manufacturing (CAM) solutions, making it feasible to translate CAD data to the robotic controller [4,5,6]. However, SMEs are reluctant to adopt such technology due to the commercial cost and the need for trained personnel with advanced robotics knowledge.

Moreover, as it relies heavily on the component and environment CAD models, additional cumbersome calibration procedures are required to compensate for as-built part tolerances and to locate the workpiece in the environment accurately. Nonetheless, path planning generated from Visual Servoing (VS) guides the robotic arm with respect to a target object based on nominal CAD and on visual feedback vs. approaches that are characterized by a high computation load to deploy real-time image processing algorithms and a requirement for nominal CAD models [7]. The use of specialized optical, tactile, or proximity sensors to fully automate the path-planning process is defined as sensor-guided programming. On the one hand, this increases the cost of robotic systems; on the other hand, the sensors are susceptible to errors due to specific material properties and require sophisticated software tailored to the application requirements [2,8].

**Figure 1:**
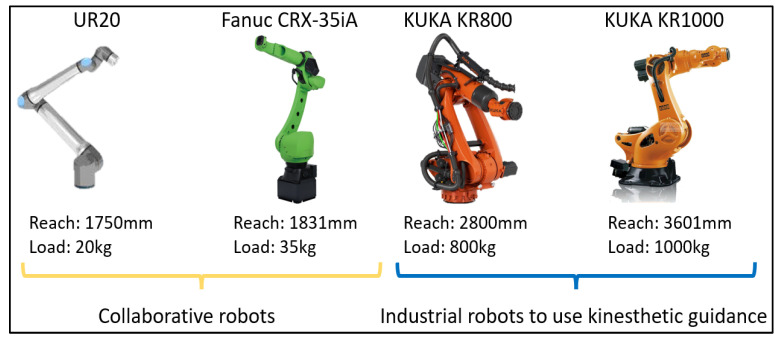
Comparison of collaborative robotic arms and industrial manipulators in terms of reachability and load capacity [14,15,16]. These industrial arms can support the kinesthetics concept for path planning, transforming these robots into collaborative entities.

Currently, industrial manipulators that perform heavy-duty tasks, such as welding, inspection, milling, painting, or pick-and-place operations, predominantly use OP approaches to teach robotic paths, remaining inflexible to complex-shaped components and with limited adaptability to fabrication variations. An intuitive way of robot programming originates from the concept of collaborative robots. Over recent years, collaborative robots have gained popularity in the global manufacturing sector owing to their design, as their joints are equipped with force–torque sensors, offering the ability to sense human interaction and work alongside human operators sharing the same working space [9,10]. This hardware capability leads to a new robotic programming approach called hand-guiding or kinesthetic guidance [11,12,13]. This method is described as a walk-through programming approach where the operator manually moves the manipulator’s end effector to the points of interest while storing the robot configuration in the points of interest. At the same time, singularities are identified and collisions with possible fixtures are revealed. However, these collaborative robots from brands such as KUKA, FANUC, and Universal Robots are designed for low-duty tasks and still lack the benefits of industrial robots such as high reachability (>2 m) and high payload capacity (>35 kg), which are commonly required for heavy industrial manufacturing sites. This can be seen in Figure 1, which demonstrates a comparison of collaborative robots and industrial 6 Degrees of Freedom (DoF) industrial manipulators regarding reachability and load capabilities.

Considering these challenges, there is an imperative need to transform current industrial robotic manipulators into collaborative entities able to be programmed with kinesthetic guidance to (i) minimize downtime between tasks, (ii) cope with high variability in components specification, and (iii) accelerate the production process from start to finish and, at the same time, not to be limited to the restricted loading and reachability constraints of collaborative manipulators.

One particular technology that has been rapidly developed, and is part of the Industry 4.0 revolution that would benefit from this transformation would be metal Additive Manufacturing (AM) and, particularly, Wire–Arc Additive Manufacturing (WAAM) based on direct energy deposition [17]. This technology offers commercial benefits to the aerospace and defense sectors, in particular, due to the ability to manufacture lightweight, complex geometry and large-volume components [18]. Following fabrication, the quality assurance of these components is conventionally performed by Non-Destructive Testing (NDT) with manually deployed methods such as Ultrasound Testing (UT) [19], Eddy Currents (ECs) [20,21], and X-ray [22]. Recent developments have also made in-process inspection possible, robotically deploying UT during manufacturing with the potential to detect defects in real-time, minimizing production costs and increasing the overall production quality [23]. Though WAAM robotic path planning for fabrication is generated through OLP based on the CAD models of the end components [24], robotic inspection still employs OP due to the complex net-shaped structure and the high mix of linear and arc motions required for volumetric structure evaluation. It is evident that the introduction of kinesthetic guidance for the inspection of complex-shaped and large components utilizing current robotic manipulators, with higher reachability and load capability, would minimize the time-consuming task of path planning and would reduce the training costs for robotic operators. This would, in turn, lead to the increased adoption of these digital technologies by SMEs, increasing their global competitiveness.

### Contribution to Knowledge

This paper presents, for the first time, a novel software system for industrial robotic manipulators to realize collaborative path planning using kinesthetic guidance in real-time (at a 250 Hz interpolation rate) by utilizing a 6 DoF force–torque (FT) sensor mounted at the robot’s out-bore flange. As such, with the presented work:

- Current industrial robotic manipulators installed in HMV sectors characterized by high reachability (>2 m) and payload capabilities (>35 kg) can be utilized by humans as collaborative entities to intuitively teach the robotic path in the manufacturing ecosystem;
- All singularities that may arise and collisions with possible fixtures are realized during the path-planning process. Compared to traditional OP and OLP robotic programming, in kinesthetic path planning, a real-time motion is executed from start to end;
- The intuitive way of collaboratively performing the path planning for industrial robots achieves advanced performance over OP and OLP by decreasing the robotic programming time by 88% and 98%, respectively; (see Supplementary Materials)
- Adaptive FT motion for defects inspection is achieved through the deployed software system and the real-time feedback of the FT sensor to the kinematics generation. This feature enables the adaption of the robotic motion to complex curvatures by generating the kinematics in real-time (250 Hz) and avoiding the distortion of the motion trajectory profile due to the parallel deployment of FT sensory corrections upon the main robotic motion;
- Compared to the previous work of the authors in [23,25], the presented work achieves dynamic adapting of the robotic motion during UT inspection to the overbuild surface features of a complex near-net-shaped WAAM component and identifying the embedded defects with a Signal-to-Noise ratio (SNR) of 10 dB.

The capabilities of the developed system are demonstrated through a KUKA KR90HA with a reachability of 3.1 m and a payload of 90 kg [26]. Kinesthetic guidance teaches the robotic path for defects inspection on a complex near-net-shaped build metal WAAM component, specifically designed and manufactured to represent typical features often found in aircraft landing gear components [18]. Following the robotic path generation, the same software system is used to conduct post-manufacturing NDT through a high-temperature dry-coupled UT roller probe [27,28] by dynamically applying FT control for optimum ultrasound coupling. The demonstrated kinesthetic guidance approach is compared with traditional OP and OLP programming approaches, proving superior in terms of time, the number of teaching points required, and robustness. As such, it is envisaged that the concept presented in this work makes it possible to retrofit current industrial manipulators, thereby improving flexibility and competitiveness and securing human resources by working collaboratively with robotics with wide applicability to HVM sectors.

## 2. Software System Architecture

The adaptive Non-Destructive Evaluation (NDE) UT inspection of a complex-shaped WAAM component demonstrated in this work utilizes (i) the kinesthetic guidance for intuitive path planning, described in this section, and (ii) the real-time kinematics generation, which was developed in previous work [25]. In parallel, the need for an adaptive FT control approach applied in parallel with the kinematics generation for optimum ultrasound coupling is described.

### 2.1. Real-Time Kinesthetic Guidance

The kinesthetic guidance for path planning is implemented through the Robotic Sensor Interface (RSI) developed by industrial robot manufacturer KUKA [29] in conjunction with the feedback of a 6 DoF FT sensor. This framework allows the influencing of the robot motion in deterministic real-time intervals purely based on sensory input, resulting in an adaptive motion that can execute at an interpolation cycle rate of 4 ms for KUKA Robot Controller (KRC)-4 or 12 ms for KRC-2-based robots. In developments [30,31] based on RSI, different processes were explored, allowing real-time adaption during NDE inspection of a composite wing panel, utilizing OLP to generate the path planning. Similar frameworks for external real-time control are also available and were developed by other robotic manufacturers, such as Dynamic Path Modification (DPM) by Fanuc [32] and External Guided Motion (EGM) by ABB [33]. Hence, the kinesthetic guidance methodology described herein can be applied to a wide range of industrial manipulators.

**Figure 2:**
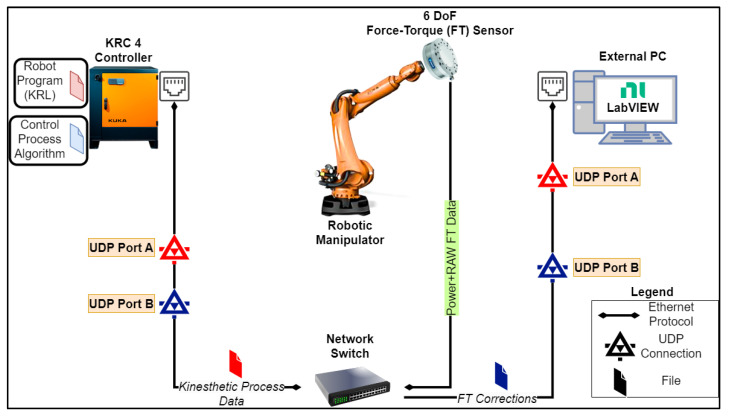
Communication interface for kinesthetic guidance and real-time kinematics generation based on the RSI protocol between external PC, KRC, and FT sensor. The external target updates, cyclically, the control process algorithm in the KRC4 controller.

Real-time kinesthetic guidance requires a control process algorithm to be executed cyclically at the robot controller and a User Datagram Protocol (UDP) connection between the controller and an external control target (e.g., PC, NI cRIO, NI PXI, Raspberry Pi, or LattePanda). This connection allows exchanging of process data, such as FT measured values, robot positional corrections, and Boolean input/outputs [25]. Figure 2 describes the communication interface for kinesthetic guidance between the external PC and the robot controller.

#### 2.1.1. Real-Time Control Process Algorithm

**Figure 3:**
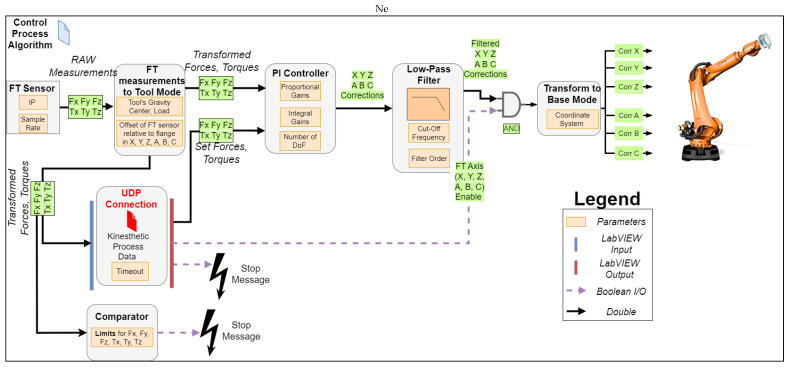
Real-time control process algorithm for kinesthetic guidance path planning describing the cyclical flow of process information between FT sensor current measurements, setpoint forces and torques, LabVIEW external control program, and generated robot positional corrections.

The construction of the control process algorithm, which executes at a 4 ms interpolation cycle rate in the KRC-4, consists of different modules, which can be found as functions on the toolbox of the RSI package. These range from signal processing (e.g., sine generator, Proportional–Integral (PI) controller, and filters), logic (e.g., or, not, and exor), and communication (e.g., ethernet UDP/Tool Centre Point (TCP) connection and digital IO) to math (e.g., min–max and multiplication). In addition, the KUKA FT package comes with additional functions such, as integrating an ATI FT Sensor, transforming FT raw measurements to other coordinate systems, a PI controller to generate positional corrections, and a load determination module for the end effector. The interconnection and the flow of process information between these modules realizing the kinesthetic path teaching can be seen in Figure 3. 

In each interpolation cycle, the FT-measured forces and moments are transformed relative to the end-effector TCP. This transformation is based on the tool’s center of gravity, load, and the FT sensor’s origin transformation to the robot’s flange. This stage is essential to realize and interpret FT measurements from the TCP accurately to the sensor’s origin while the operator drives the end effector to the points of interest. The transformed forces and moments (e.g., FT_Meas.Fx and FT_Meas.Tz) are fed to the LabVIEW interface through a UDP connection in port A, as can be seen in Figure 2. The same UDP port is used by the LabVIEW application to write the setpoints for all the FT values (e.g., FT_Set.Fx and FT_Set.Tz) to the PI controller.

During the kinesthetic teaching phase, all the force and moment setpoints are set to zero; hence, the motion of the end effector will oppose any externally applied force and/or torque, allowing for the operator to hand guide the end effector to a desired point in the workspace. In order to maintain a zero pre-set force and torque, the PI controller produces a positional correction in the tool coordinate frame. These positional corrections are generated based on the transformed FT values and the set proportional and integral gains. The generated corrections pass through a low-pass filter to remove any outliers from the sampling of raw measurements and produce a smooth motion for the end effector. Before these corrections are applied, they are multiplied with a Boolean array (1, 0), set from the LabVIEW Graphical User Interface (GUI), which allows independently enabling the individual DoFs in the cartesian space.

For example, an application requires a 2D plane surface to be covered by the end effector. In this case, a 6 DoF motion might not be required, and disabling the control of some axes could make it easier to maintain the end effector normal to the surface. Once these corrections are transformed into the base coordinate motion frame, they are applied to the robot motion trajectory. Furthermore, positional encoding takes place within the same interpolation cycle based on [25], and the robot position is transferred to the LabVIEW input through the kinesthetics process data UDP connection. The following safety provisions were implemented: (i) the overall correction in each axis is constrained within maximum allowable limits in the positive and negative directions, providing a virtual box of operation, (ii) the user can stop the kinesthetic teaching by pressing a button on the LabVIEW GUI, and (iii) the force and torque measurements are continuously monitored, and the manipulator is stopped if they exceed the maximum set limits.

#### 2.1.2. FT Feedback for Adaptive Motion Control

**Figure 4:**
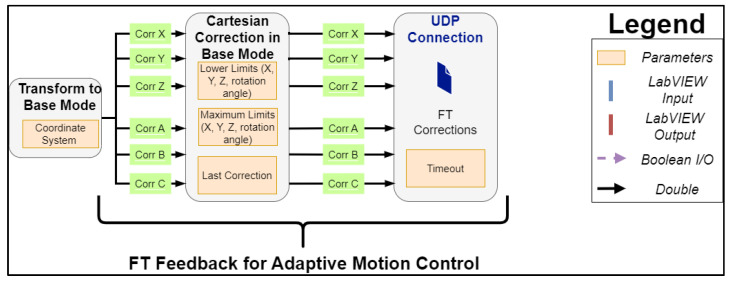
Expansion of the control process algorithm of kinesthetic path planning (Figure 3) to support the direct feedback of the FT corrections to the LabVIEW environment for adaptive motion control.

During an interpolation cycle, positional corrections in the cartesian frame of the TCP motion are produced by the LabVIEW interface based on the set acceleration, speed, and end target [25]. The PI controller of the FT sensor, which is executed in the robot controller, generates positional corrections in the same coordinate frame simultaneously; these need to be communicated in the next interpolation cycle back to the LabVIEW environment and accounted for by updating the end-target position. Thus, the motion kinematics are re-calculated on the fly, eliminating possible distortions to the generated cruise trajectory profile. To achieve this, the control process algorithm is expanded with an ethernet module, as can be seen in Figure 4, to account for the FT feedback corrections. These corrections are communicated through the UDP port B, as stated in Figure 2.

### 2.2. LabVIEW Real-Time External Control

**Figure 5:**
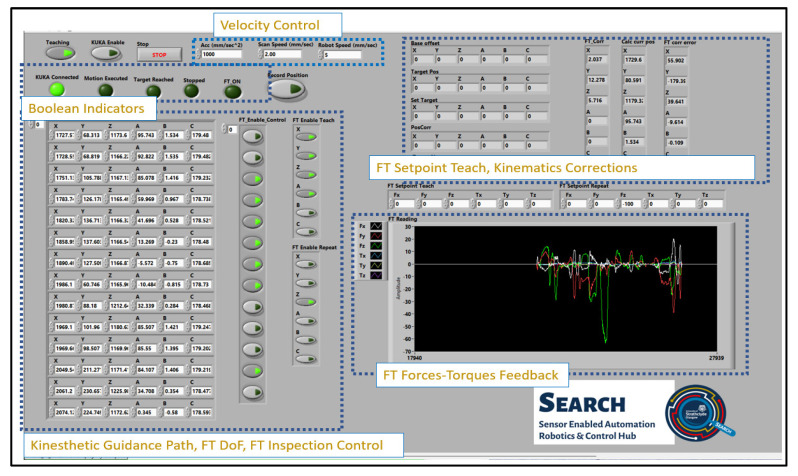
LabVIEW External Real-Time Control GUI, which handles the kinesthetic teaching and the generation of kinematics for the taught path.

The external program that controls the real-time process algorithm is developed in LabVIEW, and the GUI is depicted in Figure 5, consisting of several numeric, Boolean indicators, controls, and a real-time FT measurement plot that enables the kinesthetic teaching control and the kinematics generation control for the taught path [25]. Once the user initiates the KRL program in the teach pendant and full external control is given to the RSI interface, then it can initiate the LabVIEW application.

If the Boolean teaching control (top left Figure 4) is enabled, the user can drive the end effector to the points of interest linked to the nature of the robotic task. The system has the ability for the real-time continuous storing of the end-effector positions or discrete point-to-point teaching of the robotic path. The latter approach was selected for the application presented herein, as this can be directly compared with OLP and OP path programming. The individual DoF for the FT control can be enabled from the FT_Enable_Teach Boolean array, and the pre-set forces and torques are set to zero in the FT_Setpoint Boolean array. The user can save an end-effector position by pressing *Record Position*, which appends the current position to the Motion Path 2D array.

Following the kinesthetic teaching, the generation of kinematics between each taught point takes place once the KUKA Enable control is activated. The end-effector velocity and acceleration can be adjusted on the fly during the motion, while a separate numeric control sets the robot scan speed during UT inspection in accordance with the sampling rate and other acquisition parameters. The Boolean array FT_Enable_Control is generated next to the Motion Path array, allowing the user to enable the FT correction during motion, ensuring consistent contact with the specimen under inspection. Once contact is made, the UT data acquisition is automatically triggered. Additional inputs can be added in a similar manner to trigger different application-specific actions, such as turning on a welding torch or increasing the rotational speed of a milling tool. The FT_Enable_Repeat Boolean array allows the user to select which axes the FT-based correction should be applied to. Lastly, the FT_Setpoint repeat numeric control array is used to select the force and/or torque value for each cartesian axis in those points where the FT_Enable_Control is activated. The Boolean values for the DoF under FT motion control and the setpoint values are communicated cyclically with the real-time control process algorithm through the FT Axis Enable (X, Y, Z, A, B, C) Boolean array, as depicted in Figure 3.

## 3. Kinesthetic Complex Path Planning for WAAM UT Inspection

**Figure 6:**
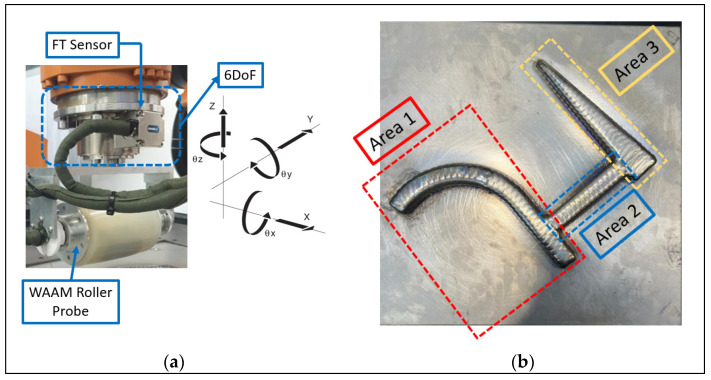
Experimental setup: (**a**) robotic setup with a 6 DoF FT sensor and a WAAM roller probe for NDE inspection mounted as an end effector; (**b**) WAAM component consisting of three sections with three embedded defects.

A UT inspection case study was carried out with a representative WAAM component, manufactured from Ti-6Al-4V of approximately a 21 mm wall height, to demonstrate complex geometry dry-coupled ultrasound NDE using the proposed kinesthetic teaching concept, as shown in Figure 6b. The dry-coupled method is utilized in this demonstration as there is no need for coupling between the ultrasound probe and the sample, which can result in contamination with subsequent passes and also can be conducted in parallel with the deposition, eliminating costs from repair activities. The manufacturing of the specimen took place in the RoboWAAM cell (Digital Factory, Renfrew, UK [34]) and was transported into the NDE cell for a volumetric inspection before feature fabrication. The 500 mm^2^ footprint complex geometry consists of features commonly found in aerospace components and can be split into three different areas: a mixed curved–linear area (Area 1), a connected linear T joint (Area 2), and a linear section with reduced width (Area 3). Three bottom-drilled holes of a 2 mm diameter were embedded 5 to 10 mm below the surface of the sample. These embedded defects simulate a lack of fusion and keyholes that commonly occur during WAAM manufacturing [35]. A KUKA KR90HA R3100 robotic arm with an FT sensor (FTN-GAMMA-IP 65, SI-130-10 Schunk) on the robot flange was utilized to demonstrate the kinesthetic path planning and adaptive UT inspection. A high-temperature dry-coupled WAAM roller probe [27,36] with a 5 MHz 64-element Phased Array (PA) transducer was mounted to the FT sensor to perform the NDE inspection, as can be seen in Figure 6a. During kinesthetic teaching, the allowed number of DoF was set to six, as can be seen in the annotated cartesian frame, providing the operator full flexibility for kinesthetic path planning over the operating area of 500 mm^2^.

Before the initiation of the kinesthetic path planning: (i) the FT sensor was calibrated by running the KUKA load determination, estimating the end effector’s load and center of gravity relative to the origin of the FT sensor (Table 1) and (ii) the proportional and integral gains for the PI controller of the FT sensor were set, as given in Table 2, and (iii) the second-order low-pass filter was set with a 5 Hz cutoff frequency to smooth the raw FT measurements. These values were selected based on previous experimental trials with an aim of producing a smooth motion without vibrations during the kinesthetic path planning and inspection process.

**Figure 7:**
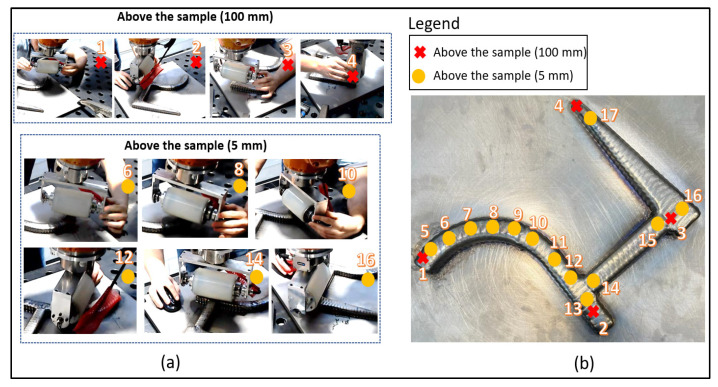
Kinesthetic teaching: (**a**) Four points recorded 100 mm above the workpiece, which are the starting, between, and endpoints during the three areas of inspection. Thirteen points were recorded by manipulation of the end effector 5 mm above the specimen, and at these points, the adaptive FT control was enabled to perform the UT inspection for defects; (**b**) top view of the complex-shaped WAAM component showcasing the taught positions generated from the kinesthetic path planning.

The end effector was driven from the user to the points of interest for each one of the three areas. Four clearance positions with a standoff 100 mm above the sample surface were recorded and added to the Motion Path at the start, between, and at the end of the three areas, as can be seen in Figure 7b. The other thirteen recorded positions were 5 mm above the WAAM component. These were the positions where the FT_Enable control was activated for the UT inspection, and as such, the roller probe maintained uniform contact with the sample.

**Figure 8:**
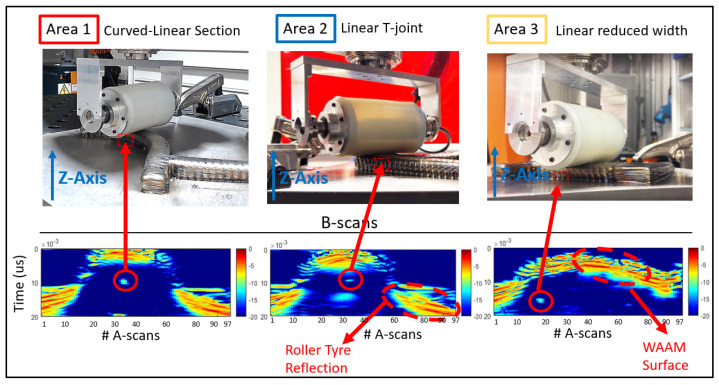
UT NDE Inspection took place following the kinesthetic guidance. The kinematics generation based on the feedback of the FT PI controller to the endtarget position adapted the motion to the overbuild features of the WAAM component.

Following the kinesthetic teaching, the adaptive UT inspection (Figure 8) was performed with the dry-coupled PA roller probe driven by a high-speed ultrasound controller LPTA (PEAK NDT). In this work, the volumetric dry-coupled inspection was conducted using conventional electronic beamforming, featuring a 32-element sub-aperture focused on the center of the deposited volume. The 33 A-scans produced by the 64-element PA were stacked to form B-scan images live during the inspection. The inspection parameters set during the inspection were 200 V of voltage and 60 dB of hardware gain.

During the inspection, a constant force of 100 N was required for dry-coupling of the probe to the sample surface and was set to the FT_Setpoint Repeat array with the direction towards the sample (negative *Z*-axis), maintaining uniform coupling. The inspection scan speed was selected to be 2 mm/s, aligning the encoded positional data of the roller probe with the generated live beamforming ultrasound data [37].

As seen in Figure 8, the feedback from the FT sensor corrections to the LabVIEW environment made it successful in adapting dynamically to the motion of the continuously varying overbuilt surface features of the WAAM component. The demonstration of this was best seen on the B-scans representing locations on the WAAM volume featuring arbitrary surface contour signals along with indications of the detected drilled holes (marked by red circles). These reflectors were located at different locations within the specimen, and a high SNR of at least 10 dB was observed at each artificial defect as well.

## 4. Quantitative Comparison

The proposed novel kinesthetic path planning concept made it possible to generate the path planning for collaborative UT inspection of the complex WAAM near-net-shaped component in 4.45 min. The integration of this software system into a KUKA KR90 industrial manipulator with a reachability of 3.1 m and payload of 90 kg to drive the end effector around the manufacturing component of the 500 mm^2^ footprint adds to the flexibility and re-usability of the robot for manufacturing and post-inspection for defect detection for large components.

The benefits of the kinesthetic guidance for current industrial manipulators used in heavy manufacturing tasks are realized through a quantitative comparison with traditional programming approaches such as OP and OLP. Two experienced users were utilized to program the robotic path using both OP and OLP robotic approaches, where the workpiece was kept in the same fixed position at all times relative to the robotic manipulator. The comparison metrics that were used were the path planning time, the number of teaching points required, the need to teach a base for motion planning, and the ability to adapt to the material overbuild of the WAAM manufacturing sample [38]. Table 3 demonstrates these results, where the proposed kinesthetic guidance approach proved robust and, overall, superior across all the comparison metrics.

The workpiece consists of three different areas; the first one cannot be defined explicitly as an arc followed by a linear line but rather a mix of a variable arc radius, adding additional time to teach more points and define smaller area linear motions when OP is utilized. Nonetheless, using OP does not bring functionality to adapt the FT control during the motion to the overbuild features of the WAAM component. As such, additional time is required to define different FT programs from the teach pendant based on the required force for uniform coupling. The teach pendant approach (OP) can take between 33 and 40 min of programming time compared to the kinesthetic approach, which takes only 4.45 min. The programming time is influenced by the experience of the robot programmer. Nonetheless, the number of taught points required can vary between 17 and 25 based on the approximation of the complex geometry by a mix of linear and arc motions and the number of FT programs for each motion.

Utilizing OLP for path planning requires an accurate design of the manufactured WAAM component, which, by the nature of WAAM manufacturing technology, deviates from the original CAD by ±5 mm, resulting in overbuild material [38]. Taking into account the need for an accurate design of the operating cell where the robot operates, a calibrated base, a fine part localization, and the end-effector TCP calibration led to a time-consuming path planning approach, which can be between 387 and 402 min based on the two experienced operators that were utilized. This includes the need to generate the CAM-compiled robot program, which requires additional cumbersome adjustments to compensate for deviations between the design and the actual workpiece. As such, this approach may be preferred for large-volume production with tight specifications but inflexible for adaption to one-off production components.

## 5. Conclusions

In this paper, the design and demonstration of a new novel kinesthetic guidance software system were presented to enable rapid human-taught collaborative inspection of complex components using traditional industrial manipulators.

The described software system was based on the feedback of a 6 DoF FT sensor in real-time intervals of 4 ms, allowing the user to drive the end effector to the points of interest and generate the path planning for complex-shaped geometries, such as the WAAM component. Following the path planning, the same system was utilized successfully to perform adaptive kinematics generation over the near-net-shaped WAAM component. For the inspection of defects, uniform coupling was achieved with a constant force of 100 N with a high-temperature dry-coupled PA roller probe. The control approach around the RSI interface of KUKA robots for real-time external control aligns with similar packages from common industrial robotic brands, such as Fanuc and ABB, enabling the proposed technology to be applied to a wide variety of industrial manipulators.

The benefits that accompany the proposed kinesthetic teaching concept were revealed through a quantitative comparison with OP and OLP programming approaches, proving that the proposed intuitive programming approach was superior overall in terms of:

- Programming time;
- Number of points required;
- Need for base calibration;
- Ability to adapt to complex shape geometries.

The programming time using the flexible kinesthetic path planning accounted only for 4.45 min. The authors estimate a significant decrease in time between 88% and 98% when compared to OP and OLP robotic teaching approaches, respectively. Moreover, the proposed concept allows the user to easily readjust the motion and redefine the points in space when the fabricated component deviates from the original CAD, which is not the case for the OLP approach. In addition, the ability to re-calculate the kinematics in real-time based on the FT feedback makes it feasible to adapt the setpoint force dynamically during the motion of the roller probe over the curved parts of the WAAM component and eliminate possible distortions to the generated trajectory profile.

Future work will seek to integrate a strategy to interpolate and recognize complex curves with the minimum number of points recorded. In addition, digital controls on the robot flange will be added to increase the flexibility during teaching as well as the ability to change the gains of the PI controller on the fly for an easy transition between teaching and the deployment of motion.

Overall, the presented research enables a collaborative and intuitive way to perform the cumbersome process of path planning for industrial manipulators. The concept originates from collaborative robots, which, due to their design, are still limited with short reachability (<2 m) and payload capabilities (<35 kg). In this way, this work can retrofit current industrial manipulators, which are designed to perform welding, metrology inspection, pick-and-place, or other heavy-duty tasks, into collaborative entities in the production line, securing human resources, and increasing production throughput.

## Figures and Tables

**Table 1:** Calibration of FT sensor based on end effector’s load and center of gravity.

Load (N)	C_x_ (mm)	C_y_ (mm)	C_z_ (mm)
25.51	5.74	−4.79	72.71

**Table 2:** Proportional and integral gains for the PI controller (Figure 3).

Proportional Gains						Integral Gains		
PFx	PFy	PFz	PTx	PTy	PTz	IFx	IFy	IFz
0.5	0.5	0.5	0.5	0.5	0.5	0.2	0.2	0.2

**Table 3:** Quantitative comparison of the proposed kinesthetic guidance approach for industrial manipulators relative to OP and OLP approaches.

Programming Approach	Number of Points (#)	Programming Time (min)	No Need for Base Calibration	Adaptability and Position Readjustment
Kinesthetic Guidance (this work)	17	4.45	**✓**	**✓**
OP	~17–25	~33–40	**✗**	**✗** (Possible readjustments but not adaptable during motion)
OLP	~285–350	~387–402 (Also required the CAM production)	**✗**	**✗** (A need to re-design the CAD and generate the CAM)

Where **✓** denotes yes and **✗** denotes no. **~** denotes an estimation for OP and OLP approaches, depending on the experience of each user utilized and the resolution required to perform the UT inspection. For OLP, the CAD-CAM products generate an increased number of points based on the set robot speed and acceleration.

## Data Availability

The data can be shared upon reasonable request.

## Supplementary Materials

The following supporting information can be downloaded at: https://www.mdpi.com/article/10.3390/s23073757/s1, The following link contains a video demonstrating the kinesthetic guidance and the adaptive UT inspection for defects: https://youtu.be/a-K5_peSG5s (accessed on 1 April 2023).
